# Symbiosis constraints: Strong mycobiont control limits nutrient response in lichens

**DOI:** 10.1002/ece3.3257

**Published:** 2017-08-11

**Authors:** Kristin Palmqvist, Oskar Franklin, Torgny Näsholm

**Affiliations:** ^1^ Department of Ecology and Environmental Science (EMG) Umeå University Umeå Sweden; ^2^ International Institute for Applied Systems Analysis (IIASA) Laxenburg Austria; ^3^ Department of Forest Ecology and Management Swedish University of Agriculture Sciences (SLU) Umeå Sweden

**Keywords:** CN stable isotopes, lichen, nitrogen, *Peltigera aphthosa* (L.) Willd., phosphorus, photosynthesis, resource allocation, symbiosis

## Abstract

Symbioses such as lichens are potentially threatened by drastic environmental changes. We used the lichen *Peltigera aphthosa*—a symbiosis between a fungus (mycobiont), a green alga (*Coccomyxa* sp.), and N_2_‐fixing cyanobacteria (*Nostoc* sp.)—as a model organism to assess the effects of environmental perturbations in nitrogen (N) or phosphorus (P). Growth, carbon (C) and N stable isotopes, CNP concentrations, and specific markers were analyzed in whole thalli and the partners after 4 months of daily nutrient additions in the field. Thallus N was 40% higher in N‐fertilized thalli, amino acid concentrations were twice as high, while fungal chitin but not ergosterol was lower. Nitrogen also resulted in a thicker algal layer and density, and a higher δ^13^C abundance in all three partners. Photosynthesis was not affected by either N or P. Thallus growth increased with light dose independent of fertilization regime. We conclude that faster algal growth compared to fungal lead to increased competition for light and CO_2_ among the *Coccomyxa* cells, and for C between alga and fungus, resulting in neither photosynthesis nor thallus growth responded to N fertilization. This suggests that the symbiotic lifestyle of lichens may prevent them from utilizing nutrient abundance to increase C assimilation and growth.

## 1 INTRODUCTION

Symbioses are defined as long‐term partnerships involving exchange of goods and division of labor between members of different Kingdoms (Douglas, 2010), which may be particularly beneficial in suboptimal environments where the partners combined physiological properties can increase the fitness of both (Boyle, Lenton, & Watson, 2011). There are many examples including lichens where symbiotic life has extended the ecological range compared to the partners’ ancestral free‐living life (Boyle et al., 2011). The benefit of symbiotic cooperation has further been suggested to be higher the more integrated the partner's physiology has become which may have reduced “selfish” alleles during evolution (Boyle et al., 2011; Douglas, 2010). However, even if a high degree of integration can explain the success of symbiotic organisms in resource poor environments, this lifestyle can be threatened by environmental changes that may favor one partner over the other. So, in view of human‐induced alterations of global nutrient cycles (Vitousek, Porder, Houlton, & Chadwick, 2010), and the significant contribution of symbiotic organisms to these cycles (Douglas, 2010; Nash, 2008), we need to know more about their responses to these stresses, such as the increase of reactive nitrogen (N_r_) in the biosphere (Bobbink et al., 2010; Fowler, Pyle, Raven, & Sutton, 2013).

Lichens have been defined as a mutualistic symbiosis between a single fungus and algal and/or cyanobacterial symbionts where the partners are more or less tightly integrated (Honegger, 1991). However, a recent discovery suggests that lichens may also involve a basidiomycete yeast located in the upper cortex (Spribille et al., 2016). One of five fungi or c. 13 500 species (ignoring the yeast) is lichenized and for a majority of these, symbiotic life appears to be ecologically obligate (Honegger, 2008). The majority form more simple crustose structures, while macrolichens with their internally stratified thalli represent the most complex vegetative structures among fungi (Honegger, 2008). About 90% of the lichens have a green algal symbiont and 10% a cyanobacterial. Some 500 lichens form tripartite associations between a fungus and both types of symbionts (Honegger, 1991); perhaps also involving a basidiomycete (Spribille et al., 2016). With the exception of photosymbiodemes where both alga and cyanobacterium are photosynthetically active (cf. Green et al., 1993), the cyanobacteria of tripartite associations are confined to internal or external cephalodia compartments providing the fungus and the alga via the fungus with combined nitrogen (N) through N_2_‐fixation (cf. Nash, 2008). The alga provides the fungus with photosynthetically reduced carbon (C), while it is not clear to what extent cephalodia are self‐supported with C (Nash, 2008).

Lichen N concentrations vary between 1 and 50 mg/g DW and are highest in lichens with N_2_‐fixation (Palmqvist et al., 2002; Rai, 1988). The N concentration of green algal lichens generally reflects habitat deposition and they can assimilate both inorganic (NH_4_^+^ and NO_3_^−^) and organic N (Dahlman, Persson, Palmqvist, & Näsholm, 2004; Gaio‐Oliveira, Dahlman, Palmqvist, Martins‐Loução, & Máguas, 2005; Johansson, Nordin, Olofsson, & Palmqvist, 2010; Palmqvist & Dahlman, 2006). Many lichens have disappeared where N deposition is high and have sometimes been replaced by less sensitive or even more N‐demanding “species”; critical loads being 10–20 kg N ha^−1^ year^−1^ depending on the particular lichen (cf. Johansson, Palmqvist, & Olofsson, 2012; Pinho et al., 2012). However, we lack a clear picture of the mechanisms behind this sensitivity and the plasticity range of different species. In addition to mere toxic effects, one possibility is that N can stimulate growth of the algal symbiont at the expense of C export to the host (Gaio‐Oliveira et al., 2005). Another is an N‐induced phosphorus (P) limitation of both or one of the partners (cf. Johansson, Olofsson, Giesler, & Palmqvist, 2011).

For the more resilient lichens, excess N may be stored in the fungus as the N‐rich amino acid arginine or in the cell wall component chitin (Dahlman, Persson, Näsholm, & Palmqvist, 2003; Gaio‐Oliveira et al., 2005; Palmqvist, 2000). This would be a way for the host to control and restrain N supply to the algal symbiont. On the other hand, an increased concentration of algal cells in the thallus may increase photosynthesis and thus be beneficial also for the fungal host (Palmqvist, 2000). Indeed, net C‐gain and growth of lichens are higher the higher the thallus N concentration across species (Palmqvist & Sundberg, 2000; Palmqvist et al., 2002) and in thalli with higher algal concentration within species (Dahlman & Palmqvist, 2003; Palmqvist & Dahlman, 2006). Still, N‐stimulated growth and expansion of the alga would only be beneficial for both partners as long as there is sufficient space in the medulla, the compartment occupied by the algal symbiont, or as long as the fungal host can grow and expand in area to provide new thallus space. See Honegger (1991) and Honegger (2008) for details on thallus morphology, anatomy, and growth. However, some studies have shown that the fungus of some lichens may rather be more limited by P than by N (Johansson et al., 2011; Makkonen, Hurri, & Hyvärinen, 2007), so even if excess N would result in more algal cells in the thallus and potentially increase the C‐gain, the host's capacity to utilize the extra N and C for hyphal growth and expansion might be more dependent on the P supply.

The foliose lichen *Peltigera aphthosa* is a tripartite association with green algal *Coccomyxa* sp. and cyanobacterial *Nostoc* sp. in external cephalodia. It has been studied extensively ranging from its N_2_‐fixation and N‐metabolism characteristics (Rai, Rowell, & Stewart, 1980, 1981) to field (e.g., Dahlman, Näsholm, & Palmqvist, 2002a; Palmqvist & Sundberg, 2000; Sundberg, Näsholm, & Palmqvist, 2001) and laboratory (Alam, Gauslaa, & Solhaug, 2015) experiments to follow and model (Dahlman & Palmqvist, 2003; Hyvärinen, Härdling, & Tuomi, 2002) its performance. *P. aphthosa* has a high capacity for growth compared to many other lichens (Alam et al., 2015; Dahlman & Palmqvist, 2003; Palmqvist & Sundberg, 2000), and there is detailed information on the photosynthetic characteristics of its *Coccomyxa* symbiont (Hiltonen, Karlsson, Palmqvist, Clarke, & Samuelsson, 1995; Palmqvist, 1993; Palmqvist, Sültemeyer, Baldet, Andrews, & Badger, 1995). It has a wide geographical distribution (Martinez, Burgazo, Vitikainen, & Escudero, 2003) and seems able to maintain a balanced CNP stoichiometry (Elser, Dobberfuhl, MacKay, & Schampel, 1996) under varying nutrient supplies (Asplund & Wardle, 2015). *P. aphthosa* can further handle mild N stress when P is available (Dahlman et al., 2002a; Sundberg et al., 2001). *P. aphthosa* is, therefore, a good candidate for testing hypotheses on how a highly integrated symbiotic association can handle variations in nutrient supply.

Compared to previous studies, we have here exposed *P. aphthosa* to a higher N load with and without P in a full factorial field experiment. We isolated the algal cells and cephalodia after the experiment to assess both thallus and the partners’ growth and nutrient statuses. The possibility that *P. aphthosa* may have two fungal partners has not been specifically dealt with since the functional significance of this is yet unknown (Spribille et al., 2016). We hypothesized that; (i) N_2_ fixation and cephalodia formation will be inhibited by N fertilization while promoting algal growth and increase photosynthesis; (ii) P fertilization will promote fungal growth and increase N_2_‐fixation; (iii) N‐mediated increase in photosynthetic capacity will result in increased thallus growth provided that the fungal host can provide space for the expanding symbiont population; (iv) the fungal host is more limited by P than by N whereby the algal symbiont will grow faster than the host when N is added in the absence of P, while a more balanced growth of the partners will resume when N and P are added simultaneously.

## 2 MATERIALS AND METHODS

### 2.1 Lichen material and transplantation

The lichen *Peltigera aphthosa* (L.) Willd was collected in mid‐May 2014 in an open and mixed Norway spruce (*Picea abies*), Scots pine (*Pinus sylvestris*), and birch (*Betula* spp.) forest northeast of Umeå, Sweden (Stomberget 63°49′4 N; 20°28′6E). The lichens were collected immediately after snow‐melt and had been covered by snow since December 2013. Two hundred and twenty healthy looking thalli without apothecia were chosen for the experiment. Their dry weight (DW) was determined to the nearest 0.1 mg after rinsing from debris and drying in darkness at room temperature. Harvest DW was determined in the same way. Parallel samples were dried for 24 hr at 80°C to correct for differences in DW before and after the transplantation due to T and RH differences in the laboratory. Initial dry weight after this correction varied from 1.1 to 0.2 g. Two hundred thalli were randomly assigned to five treatments (see below) in four blocks (10 for each treatment and block); 20 were stored at −18°C to be used as Start. All thalli to be transplanted were sprayed with water and photographed and thereafter fastened with a thin nylon thread on the underside of a cubic “fruit” basket, a 7 × 7 × 7 cm transparent plastic grid with squared openings (0.8 × 0.8 mm). The transplantation baskets were placed upside‐down in the field on 13 June 2014, and pressed into the humic layer after removal of the ground vegetation to mimic the lichen's natural growth conditions. Four thalli from each treatment and block were harvested after 81 days on 2 September; the remaining six after 117 days on 8 October. The lichens were brought to the laboratory, rinsed with water to remove any remaining fertilizer solution, photographed, dried and weighed, and thereafter stored at −18°C.

### 2.2 Field site, treatments, and microclimate monitoring

The field site was similar to the collection site; a c. 80‐year‐old boreal forest stand northwest of Umeå at Täfteträsket (Tjuvudden 64°3′5 N; 20°14′3 E) and had access to electricity for automated irrigation/fertilization. Municipal drinking water was transported from Umeå and stored in 1,000‐L tanks. The ground vegetation of small shrubs and mosses was removed from 20 circular patches (*D* = 1.5 m) distributed over the treatment area (20 × 30 m), randomly assigned to five treatments and four blocks; un‐watered control (U), irrigated control (C), irrigation with 0.037 mm NaH_2_PO_4_·2H_2_O (P), irrigation with 2.5 mm NH_4_NO_3_ (N), and irrigation with both 2.5 mm NH_4_NO_3_ and 0.037 mm NaH_2_PO_4_·2H_2_O (NP). For the C and P treatments, 0.02 mm NH_4_NO_3_ was added to mimic background deposition in the area. The δ^15^N signature of the NH_4_NO_3_ salt was −0.59‰. K_2_CO_3_ (8.8 mg/L), Na_2_CO_3_ (4.6 mg/L), CaCO_3_ (5.0 mg/L), FeSO_4_·7H_2_O (0.25 mg/L), and MnSO_4_H_2_O (0.6 mg/L) were added to all treatments following Tamm (1953) as in Johansson et al. (2011). The treatments were chosen to allow comparison with previous experiments studying the plastic responses to altered N and P of three other lichen species; *Alectoria sarmentosa*,* Lobaria pulmonaria*, and *Platismatia glauca* (Johansson et al., 2011). Treatments were in this way also significantly more intensive compared to our previous N‐stress studies of *P. aphthosa* (Dahlman et al., 2002a; Sundberg et al., 2001).

A 4‐m circular tube equipped with six mist nozzles (Micro‐Mist‐Nozzle 1371, Gardena/Husqvarna, Malmö, Sweden) positioned 0.5 m above ground was attached to a water pipe (10–30 m), a 1,000‐L tank, a garden pump, and an automated system (Gardena Watering Controller 4030, Gardena/Husqvarna, Malmö, Sweden) to distribute the irrigation/fertilization solution. Treatments were initiated on 25 June and continued daily until 8 October, distributing the solution during 1 min after dawn at 05:00 a.m. until 2 September when irrigation started at 07:00 a.m. Each patch received c. 2.5 L daily corresponding to c. 200 mm precipitation during the experiment. Up until October harvest, the N deposition amounted to 150 kg/ha in the N and NP treatments and to 1 kg N ha^−1^ in the C and P treatments, and the P deposition to 2.4 kg P ha^−1^ in the P and NP.

To compare background and fertilizer deposition, all patches were equipped with two mixed bed ionic resin capsules (PST1 capsule, Unibest, Bozeman, USA). One capsule from each patch was collected at the September and October harvests, respectively, extracted using three consecutive rinses in 10 ml 1 m KCl (30 ml total) and analyzed for NH_4_, NO_3_, and PO_4_—ions on a per capsule basis (Gundale, Sutherland, & DeLuca, 2008) (Table S2).

A data logger with sensors measuring precipitation, *T*_air_, RH, Photosynthetic Active Radiation (PAR), and lichen water content (WC) was placed in the middle of the treatment area from 7 July until 11 October (Table S1; Fig. S1). Lichen WC (Fig. S2) was measured using a separate thallus placed in one U and one C treatment patch close to the data logger. Precipitation was recorded for every 0.2 mm rain, summed and stored every 10 min. All other sensors were recorded every min, averaged and stored for 10‐min interval. See Palmqvist and Sundberg (2000) for additional details. Light (PAR) in each patch was measured at three occasions (30 July, 21 September, 11 October) and calibrated against the logger PAR sensor to yield the summed irradiance, *I*_tot_ (mol m^−2^) for each individual patch (Table S2).

### 2.3 Growth, chemical analyzes, and subsampling for assays

Initial weight and harvest weight of all thalli measured at ambient T and RH were corrected to the oven DW (see above) to determine growth of all individual thalli using Equations (1), (2). The four thalli harvested in September, and the six harvested in October from each patch were pooled to calculate the average for each treatment, block and harvest occasion.

(1) $$\text{Weight Change}\;\left(\%\right)=\frac{{\mathrm{DW}}_{\mathrm{harvest}}-{\mathrm{DW}}_{\mathrm{start}}}{{\mathrm{DW}}_{\mathrm{start}}}\times 100$$

(2) $$\text{RGR}\;\left(g\;g^{-1}\;{\text{day}}^{-1}\right)=\frac{\ln\left({\mathrm{DW}}_{\mathrm{harvest}}\right)-\ln\left({\mathrm{DW}}_{\mathrm{start}}\right)}{\Delta t}$$

Equation (2) assumes constant relative growth rate (RGR) during the measured growth period, that is the number of days since the lichens were transplanted (Δ*t*).

The thalli were randomly assigned to either of the below sets of analyzes; it was not enough material to do all measurements on every thallus.

1. First thallus from each block, treatment and harvest were used to determine specific thallus weight (STW). For this, ten evenly distributed circular samples (*D* = 10 mm) were punched from the thallus when it was hydrated, thereafter oven dried and weighed to the nearest 0.01 mg; STW expressed as g DW m^−2^ wet area.
2. A second thallus from each block, treatment and harvest were freeze‐dried, pulverized in a steel cylinder with a ball mill and aliquoted for the following analyzes; c. 10 mg was dissolved in 2 ml MgCO_3_ saturated DMSO to quantify chlorophyll as in Palmqvist and Sundberg (2002); c. 20 mg was dissolved in 1 ml 99.5% ethanol to quantify chitin and ergosterol as in Dahlman, Zetherström, Sundberg, Näsholm, and Palmqvist (2002b); c. 60 mg was dissolved in 2 ml 80% EtOH with 4 mm Hepes (pH 7.5) to extract soluble carbon (C) measured and quantified as in Palmqvist and Dahlman (2006); c. 30 mg was used for determination of amino acids as in Persson and Näsholm (2001). Total C and N, and C and N stable isotopes were measured with a mass spectrometer at a certified laboratory (Department of Forest Ecology and Management, Swedish University of Agricultural Sciences, Umeå, Sweden). This laboratory also determined total thallus P concentration after extraction in 8% H_2_SO_4_ using a Kjeldahl method (G‐189‐97 Rev. 3 multitest MT7).

3a. A third thallus from each block, treatment and harvest plus four Start thalli were used to measure CO_2_ gas exchange and photosynthetic electron transport (ETR) (see below) and thallus chlorophyll (as above).
3b. The same October harvest thalli as in (3a) were used for preparation of thin cross‐sections (see below) and thallus chlorophyll (as above).

4. Algal cells and cephalodia were isolated from a fourth thallus from each treatment and block from the October harvest (see below).

### 2.4 Isotopic composition

Both N and C isotopes are expressed and presented as their relative abundance in the material where; δ^13^C = ^13^C/^12^C isotopic ratio expressed using the VPDB scale; and δ^15^N = ^15^N/^14^N isotopic ratio expressed using the atmospheric nitrogen scale. We choose to present abundance (δ^13^C) instead of discrimination (Δ^13^C) due to uncertainties regarding the atmospheric composition of the two C isotopes during the life span of the thalli at the collection site (see Farquhar, Ehleringer, & Hubick, 1989).

### 2.5 Gas exchange and electron transport

Both CO_2_‐gas exchange and Pulse Modulated chlorophyll *a* Fluorescence to obtain ETR were adopted to assess the lichens’ photosynthetic performance. The gas exchange technique also yields information on the lichens respiration which, however, can be highly variable depending on activation procedure and the specific measurement conditions (see Sundberg, Ekblad, Näsholm, & Palmqvist, 1999), while the fluorescence technique is in‐sensitive to variation in respiration. The lichen was lightly sprayed with water and re‐activated during 12–15 hr in darkness at 15°C and 95% RH prior to measurements of dark steady‐state respiration and net CO_2_ uptake with a portable photosynthesis system LI‐6400 (LI‐COR, Lincoln, NE, USA), and ETR using an IMAGING‐PAM M‐series Chlorophyll Fluorometer (IMAG MAX/L, Heinz Walz GmbH, Effeltrich, Germany). The sample was thereafter illuminated with PAR‐light (200 μmol m^−2^ s^−1^) at 15°C and 95% RH for 1 hr after the dark period to activate Rubisco and thereafter placed in the Li‐COR cuvette covering 4 cm^2^ of the thallus. Chamber conditions; 15°C, air flow 400 μmol/s, CO_2_ concentration 400 μmol/mol, and RH 60%–70%. The activation procedure followed the same protocol as in Palmqvist et al. (2002) with the dark period aiming at reducing activation respiration as discussed in detail in Sundberg et al. (1999). The thalli were blotted with tissue paper to remove any external water prior to placing the sample in the cuvette. The samples were saturated but not oversaturated with water. Measurements started with 5 min in darkness, followed by 5‐min light (200 μmol m^−2^ s^−1^), and darkness for 5 min. The average of the two dark periods were used to determine respiration. The thallus was thereafter transferred to the imaging PAM and dark adapted for determination of ETR adopting the Light Curve procedure of the instrument. The ETR at each PAR level (0, 1, 11, 36, 56, 81, 111, 146, 186, 281, 396 μmol m^−2^ s^−1^) was calculated according to the manufacturer's manual and was corrected for differences in absorptance between samples. The light level used when measuring net CO_2_ uptake was above light saturation for the Start thalli and slightly below light saturation for the field treated samples (see Section 3). Gas exchange rates were calculated both on an area and dry weight basis using the same STW determination method as above, punching three samples (*D* = 10 mm) from the 4 cm^2^ thallus part covered by the Li‐Cor cuvette.

### 2.6 Anatomical cross‐sections

Two circular samples close to each other (*D* = 10 mm) were punched from three sections of each thallus (lobe tip, center, and between tip and center). One sample for chlorophyll determination while the other was embedded in Tissue‐Tek OCT 4583 Compound (Sakura Finetek, Torrance, CA, USA) to prepare thin cross‐sections as in Johansson et al. (2011). Ten frozen (−20°C) and 20 μm thin sections were sliced with a microtome, transferred to glass slides, and covered with 50% glycerol and photographed. The three “best” cross‐sections from each section of each thallus were selected for image analysis; *that is* those with the fewest air bubbles and other obstructions and where the fungal hyphae at least partly remained under the algal layer. The medulla hyphae of this lichen are relatively loosely attached to the algal *Coccomyxa* cells and were easily disrupted during slicing. Algal layer thickness and the ratio of algal layer versus cortex thickness were obtained using the straight‐line measurement of IMAGE‐J (Rasband, W.S., US National Institutes of Health, Bethesda, MD, USA). Three measurements were made from each photo and pooled to an average, and the three averages were pooled to an average for each thallus.

### 2.7 Isolation of Coccomyxa cells and cephalodia

The alga and cephalodia were isolated from the same thallus starting by excising as many cephalodia as possible with a sharp razor blade, excluding the very smallest ones. The cephalodia were freeze‐dried and weighed to determine their mass and aliquoted for measurements of total C and N, C and N stable isotopes, and P. For P we had to pool the material from all four blocks. Cephalodia area from the same thalli was determined from the harvest photographs using IMAGE‐J. After removal of cephalodia, the rhizines and fungal hyphae underneath the algal layer were gently scraped away. The remaining material was homogenized with a mortar and pestle and filtered through a nylon mesh. The algal cells were isolated and concentrated from the filtered homogenate in isotonic sorbitol‐Hepes buffer (pH 7.5) using 80% Percoll^®^ gradient centrifugation. The resulting suspension was washed in Tween 20 and sonicated at 40 kHz for 1 min and again centrifuged at 500 × *g* for 5 min. This treatment was repeated five times to avoid any contamination of the isolate from buffer or Percoll (Gasulla, Guera, & Barreno, 2010). The final algal pellet was freeze‐dried and aliquoted for measurements of total C and N, C and N stable isotopes, and chlorophyll. Depending on treatment ca 20–30 mg dry weight of algal cells was obtained from 500 mg dry lichen thallus, so there was not enough material for additional measurements.

### 2.8 Statistics

Differences between U and C for the estimated parameters were tested with a Student's *t* test. Differences between the four different fertilizer treatments (C, N, P, NP) were then analyzed with a full factorial ANCOVA with N and P treatment and harvest time as factors and light exposure as covariate. Other regressions are specified in the respective figure legend. The analyzes were performed using the statistical packages STATISTIX 7 (Analytical Software, Tallahasee, FL, USA) and SigmaPlot 12.5 (Systat Software Inc, London, UK).

## 3 RESULTS

The U treatment capsules (reflecting background deposition) in average contained five times more P compared to the C and N treatments. The P and NP treatments had received a 15‐fold higher P exposure compared to U. The U and C treatments had received a similar N deposition from background or irrigation, respectively. The N and NP treatments had received 100–150 times more N compared to P and C (Table S2).

Six of the measured responses and variables were significantly different in the rainwater control (C) compared to the un‐watered control (U) (Table 1). As expected, growth was significantly higher in C compared to U (Table S3) as lichens need water for photosynthesis, metabolism, and subsequent growth (cf. Alam et al., 2015; Palmqvist, 2000), and the wet active time was indeed significantly increased by the artificial watering (Fig. S2). The specific thallus weight (STW) was also different between the C and U treatments (Table 1) due to a lower STW in U at September harvest (Table S3). Chitin, relative cephalodia mass and area, and δ^15^N were higher in C (Table S3), indicating a higher N_2_ fixation activity just by adding water and prolonging wet active time. The weight‐based chlorophyll concentration was lower in C compared to U (Table S3) indicating dilution of algal cells or a lowered cellular Chl concentration, expressed also when related to thallus area (not shown but use STW to calculate this from Table S3). In the following, all responses to N and/or P are compared with the C treatment.

**Table 1:** Output of a Two‐Sample *t* test testing the rainwater control (C) versus the un‐watered control (U)

Variable	*F*	*p*	*df*
δ^15^N thallus	4.37	.035	7
Chitin thallus	3.89	.047	7
Chlorophyll thallus	4.07	.042	7
Cephalodia weight	10.51	.042	3
Thallus weight change	19.31	<.001	7
Specific thallus weight	27.66	<.001	7

Data shown for responses and variables when the two treatments were different for *p *≤* *.05.

**Figure 1:**
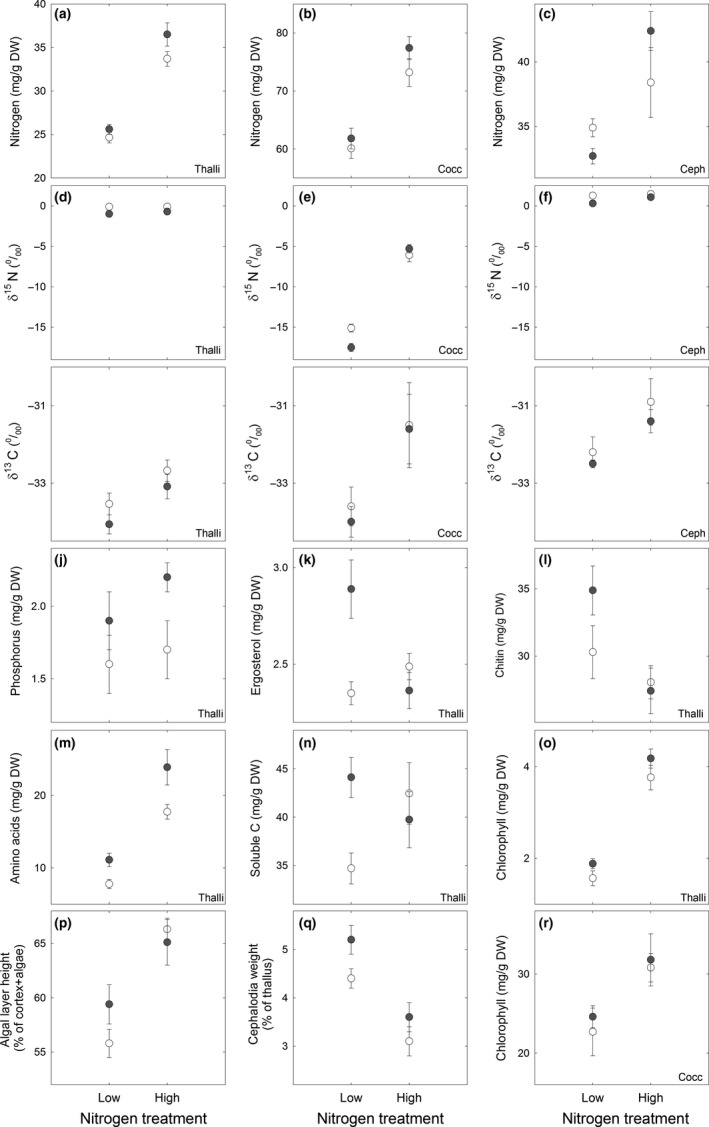
Concentrations of the measured variables and some other characteristics of whole thalli (a, d, g, j–q), newly isolated *Coccomyxa* cells (b, e, h, r), or excised cephalodia (c, f, i) in the four treatments; rainwater control (C—low‐N unfilled circle), phosphorous (P—low‐N gray circle), nitrogen (N—high‐N unfilled circle) and nitrogen plus phosphorous (NP—high‐N gray circle). Thallus data were pooled from the four blocks and both harvest occasions to an average ± 1 *SE* for *n* = 8. Data for algal cells and cephalodia were obtained only from October harvest to an average ± 1 *SE* for *n* = 4. See Section 2 for additional details. Note that the *y*‐axis scales do not start from “zero” in any of the subfigures. *y*‐axis scales are different in Figures a–c, while the same *y*‐axis scale was chosen for the δ^15^N data from thalli (d), the alga (e) and cephalodia (f) so significant treatment effects are not obvious; see Table 2 for statistics

**Figure 2:**
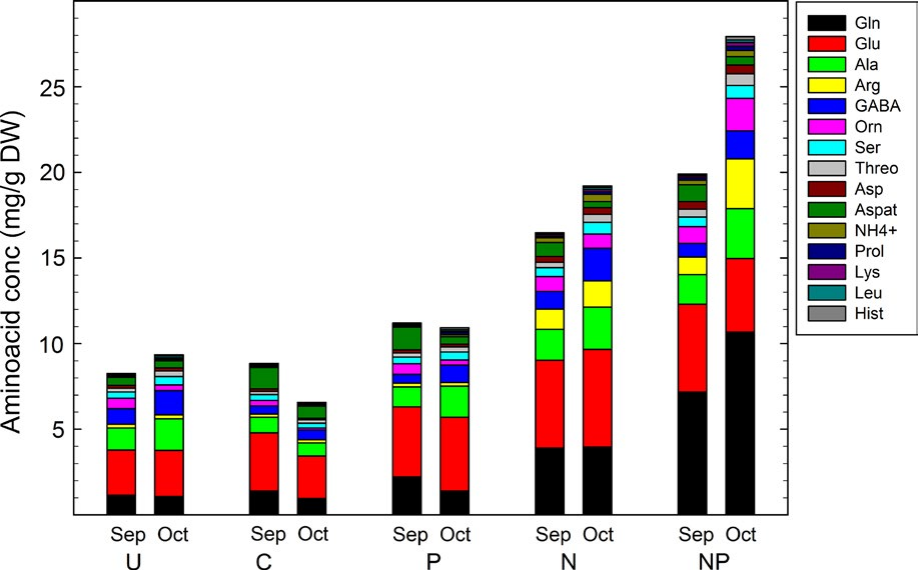
Thallus concentrations of NH_4_^+^, the 14 most abundant amino acids and GABA in the five treatments at the two harvest occasions for *n* = 4; un‐watered control (U), rainwater control (C), phosphorous (P), nitrogen (N), and nitrogen plus phosphorous (NP)

Addition of combined reactive N in the form of NH_4_NO_3_ resulted in a higher N concentration of intact thalli, the isolated algal cells and cephalodia (Table 2). The N concentration was c. 40% higher in the thalli and c. 25% higher in the alga in the N and NP treatments; *that is* high‐N compared to the two low‐N treatments; C and P (Figure 1a–b). The alga had a c. twice as high‐N concentration compared to both cephalodia and whole thalli in all treatments, and cephalodia somewhat higher than whole thalli (Figure 1a–c; Table S3). The thallus amino acid concentration was doubled in high‐N compared to low‐N (Table 2; Figure 1m), with the highest content in the combined NP treatment at October harvest (Figure 2; Table S3), thus increasing significantly more than the N content per se. Glutamine was the most abundant amino acid in the low‐N treatments, while glutamate was the most abundant in the combined NP treatment. Arginine, which has the lowest CN ratio of the amino acids, was fivefold to 10‐fold higher in high‐N compared to low‐N, and the nonproteinogenic ornithine was also significantly higher in high‐N (Figure 2). Despite of the higher thallus N and amino acid concentrations, the fungal nitrogenous cell wall component chitin was lower in high‐N compared to low‐N (Tables 2 and S3, Figure 1l).

**Table 2:** Output of the ANCOVA testing the differences between the four treatments; rainwater control (C), rainwater with phosphorous (P), rainwater with nitrogen (N), and rainwater with both N and P (NP), using N, P and harvest month as factors and summed light exposure as a covariate

Variable	N	P	NP	Month	Light
	*p*	*p*	*p*	*p*	*p* (student's *T*)
Nitrogen_tot_					
Thallus	**<.001**	**.020**	.230	.139	—
*Coccomyxa*	**<.001**	.161	.548	—	—
Cephalodia	**<.01**	.605	.082	—	—
δ^15^N					
Thallus	.395	**<.001**	.325	**.004**	**.001** (−3.79)
*Coccomyxa*	**<.001**	.161	.548	—	—
Cephalodia	.092	**.023**	.288	—	—
Carbon_tot_					
Thallus	.444	.776	.817	.447	—
*Coccomyxa*	.120	**.025**	.670	—	—
Cephalodia	**.021**	.304	.176	—	—
δ^13^C					
Thallus	**.002**	.220	.966	.306	**.021** (2.49)
*Coccomyxa*	**.012**	.738	.871	—	—
Cephalodia	**.009**	.309	.854	—	—
C:N					
Thallus	**<.001**	**.027**	.701	.701	—
*Coccomyxa*	**<.001**	.389	.614	—	—
Cephalodia	**.003**	.801	.076	—	—
Phosphorus					
Thallus	.334	**.031**	.692	.971	—
Ergosterol					
Thallus	.062	**.047**	**.003**	.413	—
Chitin					
Thallus	**.008**	.253	.134	.129	—
Aminoacids					
Thallus	**<.001**	**<.01**	.276	.106	—
Soluble carbon_tot_					
Thallus	.516	.202	**.026**	.757	—
Ribitol	.725	.198	.893	**.005**	—
Mannitol + arabitol	.668	.349	**.012**	.251	—
Chlorophyll_tot_					
Thallus	**<.001**	.070	.803	.206	—
*Coccomyxa*	**.014**	.463	.978	—	—
Chlorophyll a:b ratio					
Thallus	.158	**.039**	.182	.618	—
*Coccomyxa*	**<.001**	.371	.275	—	—
Algal layer thickness	**<.001**	.333	.109	—	—
Cephalodia weight	**.001**	**.027**	.505	—	—
Cephalodia area	**<.001**	.427	.539	—	—
Thallus weight change	.617	.127	.596	**.003**	<**.001** (4.05)
Specific Thallus Weight (STW)	.854	.141	.427	.999	—
Net photosynthesis (area based)	.674	.140	.733	.285	—
Dark respiration (area based)	.613	.628	.760	.956	—
ETR_max_	**.006**	**.036**	.669	.518	—

The table shows *p*‐values. Light was excluded as a covariate in the final output when it was found to be unsignificant (*p *>* *.05). Month and light were not included for the isolated algal cells (*Coccomyxa*) or cephalodia as data for them were obtained only from October. Bold values indicate *p*‐values <.05. Degrees of freedom = 1 for treatment, the residual = 23 for thallus contents and STW when light was included and = 24 when light was excluded; the residual = 12 for *Coccomyxa* and cephalodia, and = 151 for weight change. See methods for details on experimental design and sampling of material for the different analyzes. Concentrations of the measured responses and variables from all treatments and the two harvests are presented in Table S3.

**Figure 3:**
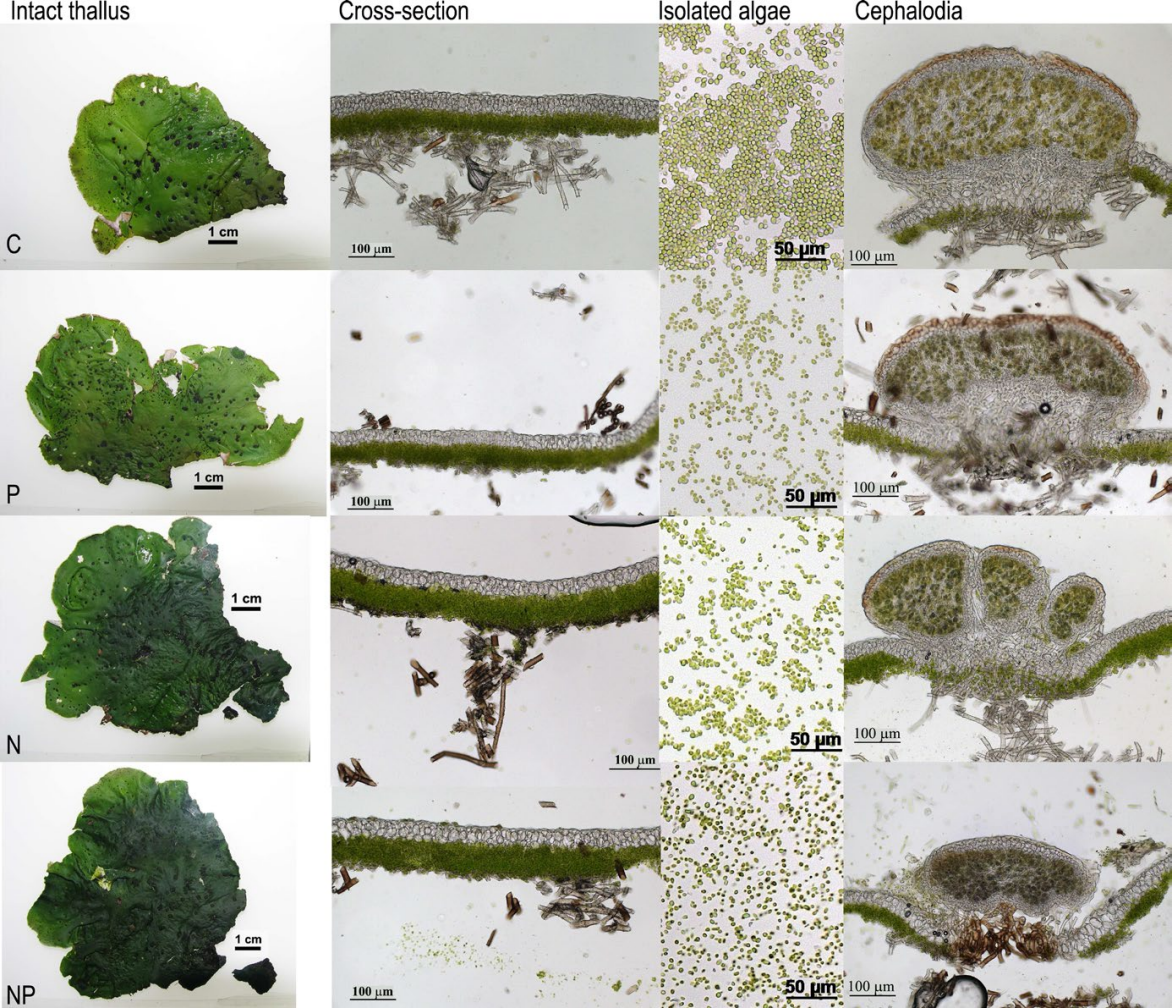
Some morphological and anatomical characteristics of the lichens at October harvest; rainwater control (C), phosphorous (P), nitrogen (N), and nitrogen plus phosphorous (NP)

**Figure 4:**
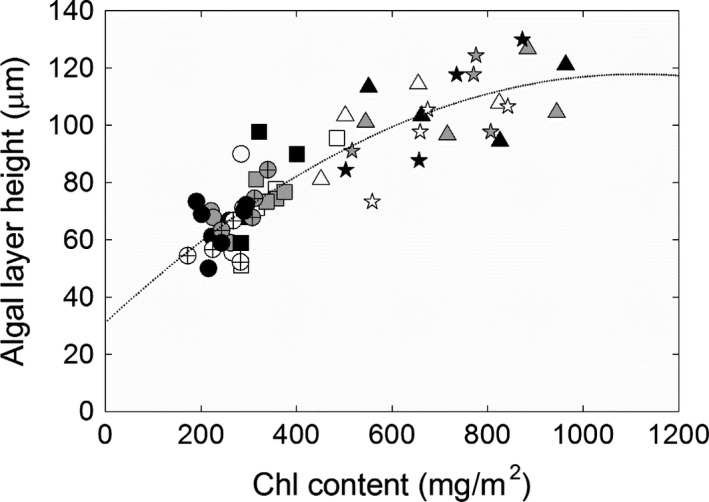
Algal layer height as a function of area‐based chlorophyll content for one thallus from each block and treatment at October harvest; un‐watered control (U—circle), rainwater control (C—circle with cross), phosphorous (P—square), nitrogen (N—triangle), and nitrogen plus phosphorous (NP—star). Data were obtained from three sections of each thallus, the lobe tip (white symbol), the center (black symbol) and between tip and center (gray symbol) as described in Section 2. All data were included to obtain the fitted curve; *y *=* *30.74 – 7 × 10^−5^ *x* ^2^ + 0.156*x*, where chlorophyll content = *x* and algal layer height = *y*;* r* ^2^ = .78

The chlorophyll concentration of whole thalli was more than doubled in high‐N compared to low‐N (Figure 1o; Table S3). Photographs of intact thalli from the N treatments revealed they were also visibly greener compared to the C and P treatments (Figure 3). A comparison of photographs taken before transplantation and after harvest also showed that all N‐treated thalli had been greener (not shown) strongly suggesting that the higher chlorophyll concentration at harvest was caused by a gradual increase during the course of the experiment. This increase was moreover manifested across the whole thallus, from the marginal and newly made lobe tips to the older central part (Figure 3; Table S3). The higher chlorophyll concentration was further a combined effect of a thicker algal layer (Figure 1p), *that is* more algal cells per unit thallus area, and a 30% higher chlorophyll concentration per algal mass in the N and NP treatments (Figure 1r). The relationship between algal layer height and area‐based thallus chlorophyll content displayed a curvilinear relationship (Figure 4), so the algal layer was not only thicker but also more densely packed with respect to algal cells and chlorophyll in the two N treatments compared to the C and P treatments. The natural abundance of ^13^C, δ^13^C, was further significantly higher in the alga in the N and NP treatments (Tables 2 and S3; Figure 1h), indicative of a decreased photosynthetic discrimination of the heavier ^13^C isotope in the thicker and denser algal layer. δ^13^C was also increased with increased light (Table 2) but was consistently relatively similar in the three fractions (alga, cephalodia, thallus) in each treatment (Figure 1g–i, Table S3). The δ^13^C values in average ranged from c. −31 to −35 for the three fractions. These δ‐values correspond to discrimination values (Δ^13^C) from 23.7 to 28.0 for an atmospheric δ^13^C value of −8‰ (see Farquhar et al., 1989) and are in the same range as for other lichens with an alga lacking a photosynthetic carbon accumulating mechanism, CCM (Máguas & Brugnoli, 1996).

Phosphorus addition resulted in higher thallus P, N, ergosterol and amino acid concentrations, C_tot_ in the alga, and weight‐based cephalodium amount (Tables 2 and S1; Figure 1). The ergosterol concentration was lower in the NP compared to the P treatment (Figure 1k), with a significant NxP interaction (Table 2). There was also a significant interaction between NxP for total soluble carbon (Table 2; Figure 1n) and the concentrations of the fungal soluble C compounds, arabitol plus mannitol (Table 2). The algal export carbon, ribitol, had a’ 30‐ to 50‐fold lower concentration compared to arabitol plus mannitol (Table S3) and was only affected by harvest month being highest in October compared to September across all treatments (Tables 2 and S3). The similar concentrations of these three compounds across treatments imply a continued export of C from alga to fungus also in the two high‐N treatments.

The δ^15^N value was above zero for the cephalodia in all treatments (Figure 1f). For the whole thallus, the value was similar as for cephalodia in each respective treatment although being slightly more negative (Figure 1d), while the alga had by far the most negative δ^15^N value (Figure 1e). δ^15^N in both cephalodia and whole thalli was lower in the P treatment compared to the C treatment (Tables 2 and S3). The δ^15^N value of the thalli also decreased with increasing light and displayed a significant interaction with harvest month (Table 2). High‐N resulted in a higher δ^15^N abundance in both alga and cephalodia (Table 2; Figure 1e–f) while lowering the amount of cephalodia both in proportion to thallus weight and area (Tables 2 and S3).

**Figure 5:**
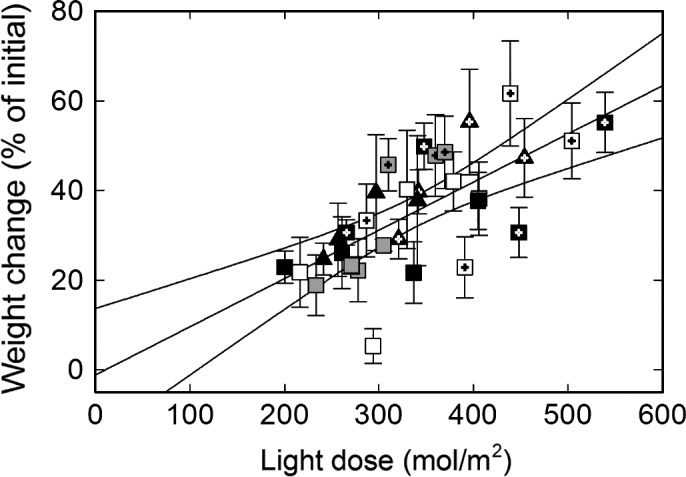
Average weight change as a function of accumulated light dose for the thalli from each block, treatment and harvest; C (white square), P (gray square), N (black square), NP (black triangle). Symbols with a cross represent October harvest. The U treatment is not included in the figure. The linear regression yielded the following equation *y *=* *0.107*x* − 1.177, where light dose = *x* and weight change = *y*;* r* ^2^ = .47. Regression displayed with a 95% confidence interval

**Figure 6:**
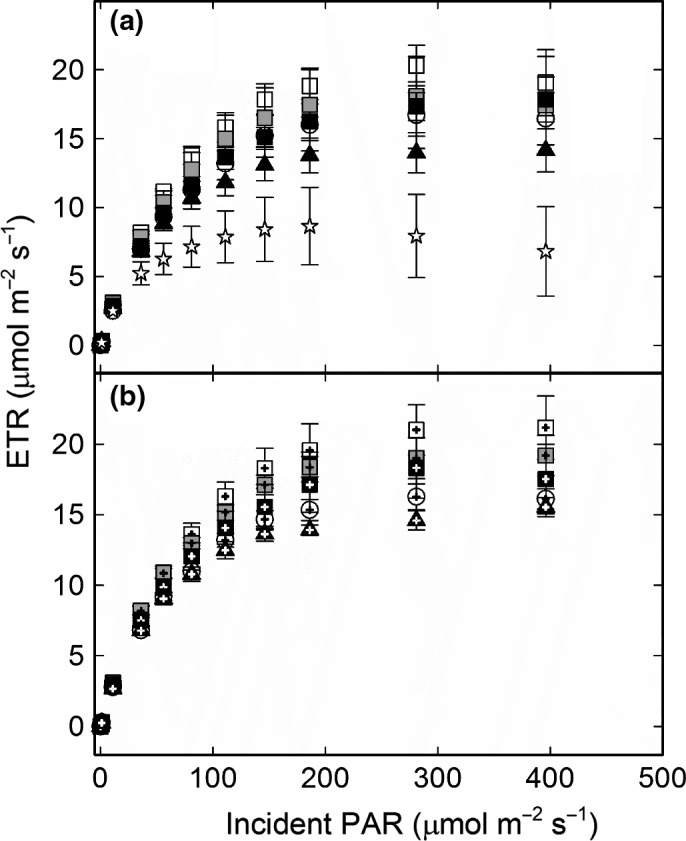
Photosynthetic electron transport rate (ETR) as a function of irradiance (PAR) after harvest in September (a) and October (b) in the five treatments; un‐watered control (U—white circle), rainwater control (C—white square), phosphorous (P—gray square), nitrogen (N—black square), and nitrogen plus phosphorous (NP—black triangle). Each curve represents the average ± 1 *SE* for *n* = 4, one thallus from each block and harvest occasion. Four Start thalli (white star) are included in (a)

**Figure 7:**
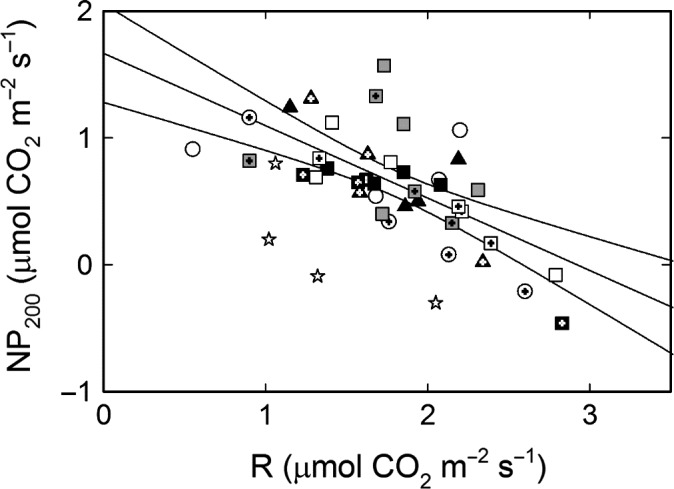
Light saturated Net CO_2_ uptake (NP) at PAR = 200 μmol m^−2^ s^−1^, and respiratory CO_2_ efflux for one thallus from each block, treatment and harvest; U (white circle), C (white square), P (gray square), N (black square), NP (black triangle). Symbols with a cross represent October harvest. The four Start thalli (white star) are not included in the linear regression yielding the following equation, *y *= −0.57*x* + 1.67, where *R* = *x* and NP = *y*;* r* ^2^ = .45. Regression displayed with a 95% confidence interval

Thallus weight gain in average amounted to c. 30% from June to September and slightly above 40% from June to October in all treatments except U (Table S3), thus without any effects of N or P (Table 2). The only factor with a significant impact on the lichens’ growth was instead variation in light between the 20 treatment units and the two harvest occasions (excluding the U treatment) regardless of fertilization regime (Table 2; Figure 5). Both N and P had significant effects on photosynthetic electron transport (ETR) (Table 2) where P resulted in a higher and N in a lower light saturated ETR (Figure 6; Table S1). Light saturation was reached slightly above 200 μmol m^−2^ s^−1^ in all treatments (Figure 6). The average dark respiration rate and light‐saturated net photosynthesis were similar across treatments and harvest month with respiration being substantially higher than net photosynthesis (Tables 2 and S3). Both ETR and net photosynthesis were lower in the lichens at the time of collection in May compared to harvest in September and October irrespective of treatment (Figures 6, 7). This suggests that the lichen was more stressed after the snow‐covered winter period than by any of the treatments. A comparison across all measured thalli (excluding the start material from May) moreover displayed a negative linear correlation between net photosynthesis and respiration without any trends related to treatment (Figure 7), but there was no correlation between growth of these thalli and their gas exchange characteristics. The thalli with negative net photosynthesis (Figure 7) had for instance a positive weight gain (Fig. S3a), and respiration and growth were neither positively nor negatively related (Fig. S3b). Among other things this emphasizes that the summed respiratory C loss must have been lower than the summed photosynthetic C‐gain during the course of the experiment. It should also be noted that the gas exchange measurements were designed for and restricted to detect whether the observed increase in algal cells had resulted in a concomitant increase in photosynthetic capacity. Both growth rates and the gas exchange characteristics were further similar to those reported in other studies (cf. Dahlman & Palmqvist, 2003; Dahlman et al., 2002a; Palmqvist et al., 2002; Sundberg et al., 2001).

## 4 DISCUSSION

### 4.1 Minor effects on nutrient status and whole lichen growth

The lichen *Peltigera aphthosa* was highly resistant during the course of the experiments to the treatments without indications of lost vitality and neither hampered nor significantly stimulated thallus growth (Tables 1 and S3; Figure 5). At the same time, however, N caused significant effects on the partners’ physiology and biochemistry. Thallus N content was increased from 25 to 35 mg/g DW by the 100‐fold increase in N‐exposure (Figure 1) which is still within the range of thallus N values in untreated *P. aphthosa* thalli (Asplund & Wardle, 2015; Dahlman & Palmqvist, 2003; Palmqvist et al., 2002; Sundberg et al., 2001). This is in contrast to other lichens during similar conditions where thallus N may be threefold to fivefold higher in N fertilized compared to nonfertilized treatments (Johansson et al., 2011; Palmqvist & Dahlman, 2006). Further, and in contrast to, for example *Platismatia glauca* in Johansson et al. (2011) the *P. aphthosa* thalli were not visibly disintegrated by the treatments (Figure 3), suggesting that *P. aphthosa* possesses mechanisms to refrain from excessive detrimental N uptake. Still, fertilization with N and NP resulted in a doubling in thallus chlorophyll concentration and a thicker layer of *Coccomyxa* cells suggesting a higher growth rate of the alga compared to the fungus (Figures 1 3, 4; Table S3).

### 4.2 Fungal control of N and C flows under natural conditions

The significantly higher algal concentration in the thalli, and N and chlorophyll concentrations per algal biomass (Tables 2 and S3; Figures 1, 3, 4) agrees with previous studies that green algal symbionts may mainly be limited by N (Johansson et al., 2011; Palmqvist & Dahlman, 2006). This also confirms earlier studies of a tight fungal control of N flow from cephalodia to alga in this lichen under natural conditions (cf. Nash, 2008). The δ^15^N signature of *Coccomyxa* was 15–20‰ lower compared to thallus and cephalodia in the control treatment (Figure 1d–f; Table S3). Plants colonized with ectomycorrhizal fungi represents an analogous case, although not as pronounced as in the lichen, where δ^15^N can be 4–6‰ lower in the plant compared to the fungus (Hobbie & Högberg, 2012; Högberg, 1997). A much lower δ^15^N of the algal symbiont compared to the whole thallus has been reported also for a lichen without N_2_ fixation where it was speculated that this might reflect acquisition of N from different sources by the two partners (Beck & Mayer, 2012). In our case, however, we suggest that the different δ^15^N signatures of the bionts may primarily be an effect of the series of controlled and ^15^N discriminating metabolic steps from cephalodia through the fungus to the alga (Rai, 1988; Rai et al., 1980, 1981). When provided by an external N source the alga could instead shortcut this control leading to the δ^15^N signature approaching the new N source of −0.59‰ (Figure 1e).

Cephalodium dry weight and area decreased by the N treatment (Figure 1; Table S3) suggesting some loss. However, new cephalodia were still formed on the newly formed thallus margins (Figure 3). One explanation for this might be independent development of cyanobacteria on the thallus surface even if host demand for internal N may have been decreased. Another explanation is that the fungus favored the internal supply and therefore retained its N_2_‐fixing partner, and continued to supply it with reduced C. Previous authors have discussed whether the cephalodia and *Nostoc* are self‐supported with C or not (Nash, 2008). Our data strongly suggests that C was primarily supplied from algal photosynthesis because the δ^13^C signature was similar in *Coccomyxa* and cephalodia in each treatment (Figure 1; Table S3). *Nostoc* possesses a photosynthetic CO_2_ concentrating mechanism (CCM) (cf. Palmqvist, 2000), and therefore discriminate the heavier ^13^C isotope to a much lower extent than *Coccomyxa* which lacks a CCM (Palmqvist et al., 1995) so the δ^13^C value should have been higher in the cephalodia, had they been more autonomous with respect to C assimilation.

The data discussed above strongly suggest that the fungal host of *P. aphthosa* tightly control both C and N flows under natural conditions, restricting the possibility for a direct exchange of C‐ and N‐between the two symbionts. Moreover, the N supply from the fungus to the alga seems to be ample as this lichen is among those with the highest green algal and chlorophyll densities (Palmqvist et al., 2002; Rai, 1988). The latter points to an association where the alga also benefits from the “living together”. It is not surprising that the fungus has to tightly control its two partners who might otherwise be able to cheat the host and exchange goods in‐between them (Boyle et al., 2011; Douglas, 2010). The alga apparently got access to more N in the N and NP treatments. It is likely that fungal control of algal N supply was then lost as the hyphal cortex mainly consists of dead hyphae (Honegger, 1991, 2008), while it is difficult to judge whether the fungus also shifted from internal (cephalodia) supply to the external source, as discussed above. Nevertheless, the thallus N concentration was still higher in the N‐fertilization treatments, and the amino acid concentration was fourfold higher in the NP compared to the C treatment, amounting to 0.7% and 2.8% of the thallus dry weight, respectively, at October harvest (derived from data in Figure 2; Table S3). The proportion of thallus N invested in amino acids was also higher, amounting to 9%–13% of the thallus N in the NP treatments compared to 4%–5% of the thallus N in the U and C treatments. This might be a strategy to avoid toxic build‐up of ammonium although at the same time increasing the C demand.

In contrast to previous studies of *P. aphthosa* showing a positive correlation between chitin and thallus N content (Dahlman & Palmqvist, 2003; Sundberg et al., 2001), this compound was decreased by N fertilization from c. 3% to 2.5% of the thallus weight (Figure 1; Table S3). The proportion of thallus N invested in chitin was 7%–8% in the U and C treatments and reduced to 5%–6% in the N and NP treatments. The fungal plasma membrane component ergosterol was, however, not lower in the two N treatments compared to control (Figure 1; Table S3). As there was no lack of N for chitin synthesis, the supposedly increased amino acid synthesis together with more C being used for algal growth may have caused a relative C deficiency for the fungus, leading to a hampered chitin synthesis. A comparison of C present as soluble C, amino acid, or chitin derived from data in Figure 2 and Table S3 showed that C in amino acids amounted to less than 15% of the C in soluble C in the control treatments while amounting to 60% of the soluble C in the NP treatment at October harvest. This emphasizes that the fertilization‐induced amino acid synthesis indeed was a significant C sink.

### 4.3 Fungal access to C limited by increased algal C demand, light, and CO_2_ diffusion limitation

Based on previous observations within and across lichen species we expected increased photosynthesis as a result of an N‐driven increase in algal cells and that addition of P together with the N would result in increased fungal and thereby thallus growth (Benner, Conroy, Lunch, Toyoda, & Vitousek, 2007; Benner & Vitousek, 2007; Johansson et al., 2011; Palmqvist & Dahlman, 2006; Palmqvist et al., 2002). However, the light response curve of photosynthetic electron transport (Figure 6) was similar across treatments and harvest time, so despite of the higher concentration of algal cells there was no increase in photosynthesis or growth of the N‐ or NP‐fertilized thalli (Tables 2 and S3). However, *P. aphthosa* has a dense algal population already under nonfertilized conditions absorbing 90% of incident light at a chlorophyll content of c. 200 mg/m^2^ (Dahlman & Palmqvist, 2003). In this study, the chlorophyll content was fivefold higher in the N and NP treatments implying that even more of the incident light could be absorbed. Based on the high, 90%, initial absorptance this increase in chlorophyll implies a reduction of light absorbed per chlorophyll of at least 77% and a strong light limitation of the lowermost algal cells. This lack of photosynthetic response to N is analogs to that shown for Scots pine trees, for which light limitation obliterated the potential effects of an N‐induced enhanced photosynthetic capacity (Tarvainen, Lutz, Räntfors, Näsholm, & Wallin, 2016).

The denser and thicker algal layer (Figures 3, 4) also resulted in a higher ^13^C abundance (less negative values) (Tables 2 and S3; Figure 1g–i). This could be the result of increased CO_2_ diffusion limitation through the algal layer and/or increased competition for CO_2_ between the algal cells, resulting in a lower internal CO_2_ concentration which would lead to a lower discrimination against the heavier isotope (Cowan, Lange, & Green, 1992; Farquhar et al., 1989; Máguas & Brugnoli, 1996). Previous studies have shown that photosynthesis is far from saturated at ambient CO_2_ in the *Coccomyxa* alga both in isolation and when lichenized (Palmqvist, 1993), indicating that such a lowering of internal CO_2_ may be an additional explanation why photosynthesis was not increased. Another factor of importance for the lichens’ net C‐gain is respiration which can be increased in N‐rich tissue including lichens (cf. Palmqvist, 2000; Palmqvist et al., 2002). However, we did not find such a relationship in this study (Figure 7).

Finally, growth of the lichen was best correlated to the variation in light between the 20 treatment units and the two harvests, with weight gain and RGR (Figure 5; Table S3) being similar to that observed for *P. aphthosa* during similar field conditions with artificial hydration but only mild fertilization (Dahlman & Palmqvist, 2003; Sundberg et al., 2001). We may then conclude that light, rather than N or P, together with hydration regime were the main limitations. In the absence of increased photosynthesis from the growing algal population, together with more C being used for algal growth and amino acid storage of surplus N in an already light‐limited situation may then explain why the N‐fertilized thalli could not grow any faster and expand in the necessary new area to provide space for the growing algal population. We hypothesize that insufficient coordination of the partners’ responses is an underlying reason for the lack of whole lichen growth response. Hypothetically, if algal growth could have been restricted, a resulting increased N concentration and photosynthesis per algal biomass could have fueled enhanced C export to the fungus (instead of algal growth) and ultimately enhanced the growth rate (area expansion) of the thallus. Such an adaptive response is what we would expect from plants. In response to nutrient additions, plants increase leaf area and carbon gain while reducing C allocation to roots and associated C costs of nutrient uptake and respiration, resulting in a net C‐gain and enhanced growth and fitness (Franklin et al., 2012). In contrast, the response of the studied lichen appeared nonadaptive, because it did not increase net growth, neither by increasing photosynthesis (C‐gain), nor by reducing C costs (respiration).

In a more general perspective, these results may reflect an inherent limitation of symbiotic organisms (such as lichens) in their ability to optimize resource allocation among their components (partners) compared to fully integrated (nonsymbiotic) organisms, such as plants. In response to increasing nutrient availability, even light‐limited plants can obtain a net gain in biomass increment and fitness by adjusting root allocation to reduce C costs of nutrient uptake (Franklin et al., 2012). Thus, while their symbiotic strategy allows lichens to conquer very harsh environments, limited coordination among partners may limit their ability to utilize increased resource availability. However, the validity of this hypothesis beyond the study presented here remains to be tested, as does the interesting possibility that it may play a role for other resource‐trading symbioses, such as mycorrhiza and corals.

## CONFLICT OF INTEREST

None declared.

## AUTHOR CONTRIBUTION

K.P. planned and designed research and experiments, conducted fieldwork, supervised field and laboratory staff, processed, analyzed and summarized all data, and wrote the text. T.N. provided laboratory facilities and helped with analysis of data, provided comments, and added significantly to the discussion. O.F. critically scrutinized and discussed lichen carbon budgeting, theoretical concepts, and generalizations.
