# Supplementary material for: Symbiosis constraints: Strong mycobiont control limits nutrient response in lichens
Source: Ecol Evol. 2017 Aug 11;7(18):7420–33. doi: 10.1002/ece3.3257 (PMC5606882; doi:10.1002/ece3.3257)
Supplement: Supplementary file 1 [file ECE3-7-7420-s001.pdf]

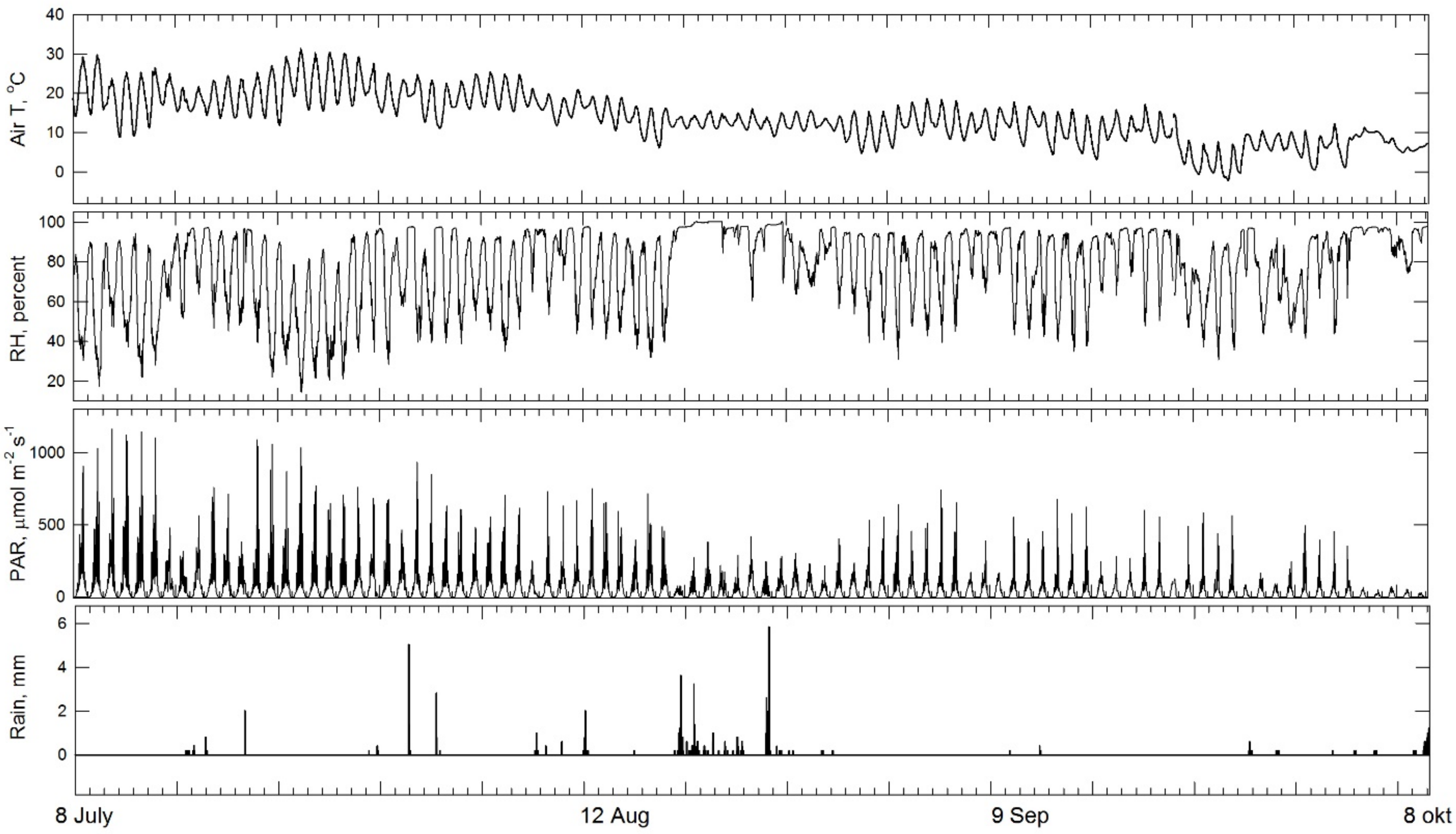

Figure S1. Air temperature, RH, PAR and precipitation at the transplantation site from July 8 to Oct 8, 2014.

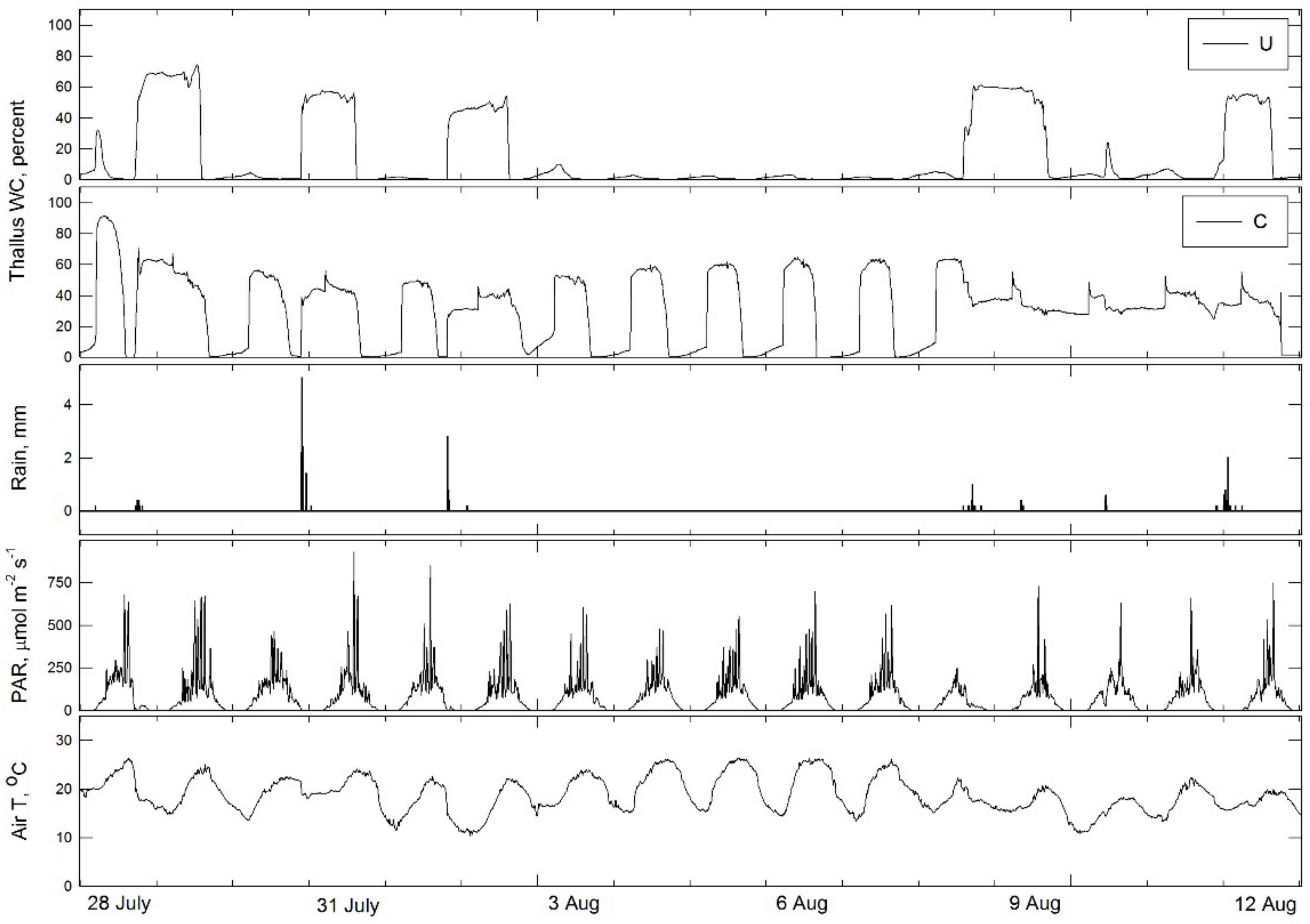

Figure S2. Lichen water content (WC) for an un-watered (U) and irrigated control (C) thallus placed among the transplanted thalli, and precipitation, PAR and air temperature during a two-week period in the middle of the experimental period (July 28 – Aug 12, 2014).

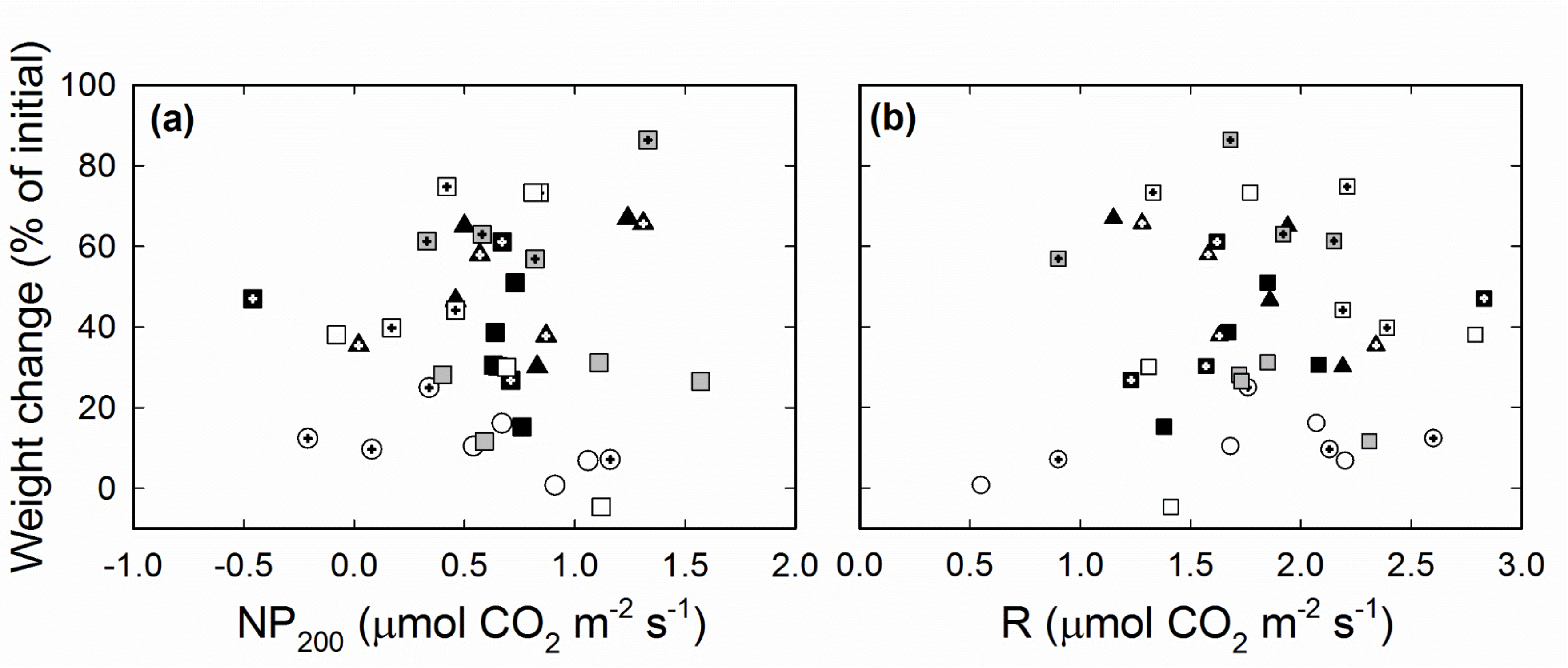

Figure S3. Thallus weight gain as a function of net CO_2 uptake (NP) at light saturation (200 μmol m^{-2} s^{-1}), 15ºC and ambient CO_2 and dark respiration for the thalli used for the gas exchange measurements; U (white circle), C (white square), P (grey square), N (black square), NP (black triangle).
