# Supplementary material for: Symbiosis constraints: Strong mycobiont control limits nutrient response in lichens
Source: Ecol Evol. 2017 Aug 11;7(18):7420–33. doi: 10.1002/ece3.3257 (PMC5606882; doi:10.1002/ece3.3257)
Supplement: Supplementary file 2 [file ECE3-7-7420-s002.docx]

**Table S1** Monthly mean, max, min or summed climate variables at the transplantation site from July 7 to Oct 11, 2014.

Month

Variable July Aug Sep Oct

Air T Mean 20.3 14.6 9.7 6.9

(*^o^C*) Max 30.5 26.4 19.7 13.9

Min 10.8 1.5 -5.6 -1.5

RH Mean 66 82 78 89

(*%*) Min 14 32 31 44

Rain Sum 23 104 7 2

(*mm*)

I_tot_ Sum 209 166 115 13

(*mol m^-2^*)
