# Supplementary material for: Symbiosis constraints: Strong mycobiont control limits nutrient response in lichens
Source: Ecol Evol. 2017 Aug 11;7(18):7420–33. doi: 10.1002/ece3.3257 (PMC5606882; doi:10.1002/ece3.3257)
Supplement: Supplementary file 3 [file ECE3-7-7420-s003.docx]

**Table S2.** Accumulated light dose and ion concentrations in the resin capsules at the two harvest occasions. Ions are expressed as *mg N L^-1^* or *mg P L^-1^* in the KCl extract. See Materials and methods for additional details.

Month Treat Block Light dose NO_3_ NH_4_ PO_4_

*mol m^-2^ mgN L^-1^ mgN L^-1^ mgP L^-1^*

Sep U 1 330 1.07 0.25 0.09

2 379 1.18 0.3 0.12

3 294 1.45 0.38 0.44

4 216 1.16 0.27 0.04

C 1 330 1.29 0.74 0.02

2 379 1.5 1.25 0.01

3 294 1.22 0.68 0.02

4 216 1.18 0.91 no data

P 1 271 1.04 0.46 2.73

2 278 1.32 0.57 2.83

3 305 1.09 0.51 1.91

4 233 1.13 0.5 2.73

N 1 405 58.85 68.54 0.03

2 261 48.12 57.81 0.06

3 337 59.19 68.88 0.05

4 200 42.58 50.19 0.10

NP 1 341 114.23 121.85 3.21

2 297 113.88 114.23 2.96

3 241 94.85 91.04 2.48

4 257 73.38 67.85 1.97

Oct U 1 454 1.54 0.36 0.13

2 396 1.71 0.44 0.18

3 321 2.09 0.55 0.63

4 342 1.67 0.39 0.06

C 1 439 1.86 1.07 0.03

2 504 2.17 1.81 0.02

3 391 1.76 0.98 0.03

4 287 1.71 1.31 no data

P 1 539 1.5 0.67 3.94

2 348 1.9 0.82 4.09

3 448 1.58 0.74 2.76

4 266 1.63 0.72 3.94

N 1 454 85 99 0.04

2 396 69.5 83.5 0.08

3 321 85.5 99.5 0.07

4 342 61.5 72.5 0.15

NP 1 360 165 176 4.63

2 370 165 165 4.28

3 406 137 132 3.58

4 310 106 98 2.85
