# Supplementary material for: Symbiosis constraints: Strong mycobiont control limits nutrient response in lichens
Source: Ecol Evol. 2017 Aug 11;7(18):7420–33. doi: 10.1002/ece3.3257 (PMC5606882; doi:10.1002/ece3.3257)
Supplement: Supplementary file 4 [file ECE3-7-7420-s004.docx]

**Table S3.** Average values±1SE for n=4 of measured parameters in homogenized whole thalli, isolated *Coccomyxa*, and excised cephalodia in the five treatments; un-watered control (U), rainwater control (C), phosphorous (P), nitrogen (N), and nitrogen plus phosphorous (NP) and the two harvest occasions. For the cephalodia all material from the four blocks had to be pooled for quantification of phosphorous. See Materials and Methods for sampling and measurement details.

Treatment

Variable Sample ID U C P N NP

Nitrogen_tot_ (*mg g^-1^ DW*)

Thallus_Sep_ 27.3±1.1 25.3±0.5 26.4±0.7 32.4±0.2 34.2±0.2

Thallus_Oct_ 26.7±0.1 24.0±1.1 24.8±0.8 35.0±1.4 38.8±1.5

*Coccomyxa*_Oct_ 63.3±0.7 60.1±1.7 61.8±1.8 73.2±2.4 77.4±2.0

Cephalodia_Oct_ 31.4±0.7 34.9±0.7 32.7±0.6 38.4±2.7 42.4±1.5

δ^15^N (^o^/_oo_)

Thallus_Sep_ -1.23±0.3 -0.25±0.2 -1.02±0.3 -0.07±0.3 -0.72±0.1

Thallus_Oct_ -1.28±0.2 0.03±0.7 -0.99±0.2 -0.15±0.5 -0.71±0.1

*Coccomyxa*_Oct_ -16.1±0.4 -15.1±0.5 -17.5±0.5 -6.1±0.8 -5.3±0.5
Cephalodia_Oct_ 1.17±0.2 1.26±0.2 0.29±0.2 1.45±0.4 1.06±0.1

Carbon_tot_ (*mg g^-1^ DW*)

Thallus_Sep_ 477±4 483±2 484±2 481±4 483±1

Thallus_Oct_ 486±2 483±6 480±3 487±2 485±2

*Coccomyxa*_Oct_ 556±4 539±2 549±2 546±4 553±4

Cephalodia_Oct_ 464±2 465±2 464±2 467±2 472±2

δ^13^C (^o^/_oo_)

Thallus_Sep_ -34.2±0.2 -33.6±0.3 -34.1±0.5 -33.2±0.3 -32.9±0.2

Thallus_Oct_ -34.1±0.4 -33.5±0.6 -34.0±0.3 -32.2±0.3 -33.3±0.6

*Coccomyxa*_Oct_ -34.9±0.3 -33.6±0.5 -34.0±0.4 -31.5±1.1 -31.6±0.9

Cephalodia_Oct_ -34.1±0.2 -32.2±0.4 -32.5±0.1 -30.9±0.6 -31.4±0.3

**Table S2 –** continued page 2

Treatment

Variable Sample ID U C P N NP

C:N (ratio)

Thallus_Sep_ 17.6±0.6 19.2±0.4 18.4±0.5 14.9±0.1 14.2±0.7

Thallus_Oct_ 18.3±0.5 20.2±0.7 19.4±0.6 14.0±0.6 12.5±0.5 *Coccomyxa*_Oct_ 8.8±0.2 9.0±0.3 8.9±0.3 7.5±0.3 7.2±0.2 Cephalodia_Oct_ 14.8±0.3 13.3±0.3 14.2±0.2 12.4±0.9 11.2±0.4

Phosphorus (*mg g^-1^ DW*)

Thallus_Sep_ 1.9±0.2 1.4±0.2 2.1±0.3 1.6±0.2 2.3±0.3

Thallus_Oct_ 2.7±0.3 1.8±0.3 1.8±0.3 1.8±0.2 2.0±0.1

Cephalodia_Oct_ 3.3 2.2 2.6 2.9 2.4

Ergosterol (*mg g^-1^ DW*)

Thallus_Sep_ 2.5±0.2 2.3±0.1 2.7±0.1 2.4±0.1 2.5±0.1

Thallus_Oct_ 2.6±0.1 2.4±0.1 3.0±0.2 2.6±0.1 2.2±0.1

Chitin (*mg g^-1^ DW*)

Thallus_Sep_ 26.2±1.3 33.1±3.1 36.0±3.0 27.6±2.1 29.3±3.2

Thallus_Oct_ 27.6±1.6 27.5±1.7 33.8±2.4 28.6±1.6 25.6±1.0

Aminoacids (*mg g^-1^ DW*)

Thallus_Sep_ 8.3±1.6 8.9±0.8 11.2±1.3 16.4±0.9 19.8±2.5

Thallus_Oct_ 9.5±0.7 6.7±0.6a 11.0±1.6 19.1±1.6 27.9±3.2

Tot Soluble C (*mg g^-1^ DW*)

Thallus_Sep_ 41±2 35±2 46±4 39±6 42±4

Thallus_Oct_ 40±1 34±3 42±1 46±1 37±4

**Table S2 –** continued page 3

Treatment

Variable Sample ID U C P N NP

Ribitol (*mg g^-1^ DW*)

Thallus_Sep_ 0.61±0.14 0.37±0.08 0.61±0.18 0.61±0.12 0.51±0.04

Thallus_Oct_ 1.50±0.29 1.07±0.56 1.70±0.72 1.12±0.28 1.94±0.70

Man+Arab (*mg g^-1^ DW*)

Thallus_Sep_ 34.5±2.5 31.8±1.6 40.5±3.2 33.3±5.2 35.4±3.3

Thallus_Oct_ 33.8±0.8 28.9±3.1 36.5±0.8 37.6±0.9 27.6±4.1

Chlorophyll_tot_ (*mg g^-1^ DW*)

Thallus_Sep_ 1.92±0.1 1.56±0.3 1.95±0.2 3.41±0.2 3.97±0.3

Thallus_Oct_ 1.88±0.1 1.57±0.2 1.82±0.1 4.12±0.5 4.39±0.3

*Centre 1.88±0.1 1.59±0.2 2.38±0.3 4.02±0.5 4.50*±0.4

*Middle 1.46±0.02 1.66±0.04 1.76±0.1 3.75±0.3 3.95±0.3*

*Margin 1.31±0.1 1.39±0.1 1.83±0.2 3.45±0.4 3.75±0.4*

*Coccomyxa*_Oct_ 21.4±1.8 22.7±3.0 24.6±1.4 30.0±1.8 31.8±3.3

Chl a:b (ratio)

Thallus_Sep_ 2.9±0.3 3.2±0.4 2.6±0.1 3.2±0.1 2.7±0.1

Thallus_Oct_ 2.7±0.2 3.4±0.2 2.8±0.4 2.5±0.1 2.7±0.1

*Coccomyxa*_Oct_ 2.9±0.3 3.1±0.2 3.1±0.1 2.5±0.1 2.3±0.1

Algal layer height (*μm*)

66±3 66±3 76±4 106±4 103±5

Cephalodia weight (*% of thallus weight*)

4.0±0.3 4.4±0.2 5.4±0.3 3.2±0.3 3.7±0.3

**Table S2 –** continued page 4

Treatment

Variable Sample ID U C P N NP

Cephalodia area (*% of thallus area*)

5.2±0.3 5.7±0.4 5.7±0.4 3.7±0.3 3.1±0.2

Weight gain (*% of initial*)

Thallus_Sep_ 5.3±1.7 27.3±8.6 23.0±1.8 27.1±3.6 32.7±3.5

Thallus_Oct_ 8.1±2.7 42.2±8.7 45.1±2.4 41.5±6.4 42.8±5.6

RGR (*mg g^-1^ day^-1^*)

Thallus_Sep_ 0.62±0.19 2.82±0.85 2.51±0.19 2.89±0.35 3.37±0.30

Thallus_Oct_ 0.66±0.19 2.95±0.53 3.19±0.15 2.98±0.39 3.03±0.32

STW (*g m^-2^*)

Thallus_Sep_ 109±6 132±1 129±4 140±9 120±7

Thallus_Oct_ 130±8 132±3 129±8 129±5 130±5

Net CO_2_ uptake (*μmol m^-2^ s^-1^*)

Thallus_Sep_ 0.80±0.12 0.64±0.25 0.92±0.27 0.69±0.03 0.76±0.18

Thallus_Oct_ 0.34±0.29 0.47±0.14 0.77±0.21 0.39±0.28 0.69±0.27

Dark resp (*μmol m^-2^ s^-1^*)

Thallus_Sep_ 1.63±0.38 1.82±0.34 1.90±0.14 1.75±0.15 1.79±0.22

Thallus_Oct_ 1.85±0.36 2.03±0.24 1.66±0.27 1.81±0.35 1.71±0.22

ETR_max_ (*μmol m^-2^ s^-1^*)

Thallus_Sep_ 17.0±2.0 20.6±1.5 18.7±2.6 18.2±1.2 14.9±1.2

Thallus_Oct_ 16.8±0.8 21.8±18.3 19.6±1.2 18.3±1.9 15.6±0.5
